# Alleviative Effects of Exopolysaccharide Produced by *Lactobacillus helveticus* KLDS1.8701 on Dextran Sulfate Sodium-Induced Colitis in Mice

**DOI:** 10.3390/microorganisms9102086

**Published:** 2021-10-02

**Authors:** Yin Liu, Shujuan Zheng, Jiale Cui, Tingting Guo, Jingtao Zhang, Bailiang Li

**Affiliations:** 1 School of Food and Biological Engineering, Zhengzhou University of Light Industry, Zhengzhou 450002, China; liuyin@zzuli.edu.cn (Y.L.); shujuanlly@126.com (S.Z.); cuijiale88@163.com (J.C.); 18437221469@163.com (T.G.); jtzhang@zzuli.edu.cn (J.Z.); 2 Key Laboratory of Dairy Science, Ministry of Education, College of Food Science, Northeast Agricultural University, Harbin 150030, China; 3 Food College, Northeast Agricultural University, Harbin 150030, China

**Keywords:** exopolysaccharide, colitis, inflammation, intestinal barrier, gut microbiota, short-chain fatty acids

## Abstract

Ulcerative colitis (UC) is a non-specific chronic inflammatory disease with lesions located in the colon and rectum. The aim of this study was to evaluate the anti-inflammatory effects of exopolysaccharide-1 (EPS-1) isolated by *L. helveticus* KLDS1.8701 on UC. The anti-inflammatory effects of EPS-1 were studied using dextran sulphate sodium (DSS)-induced UC model. In vivo results showed that EPS-1 administration significantly ameliorated weight loss, colon shortening, disease activity index (DAI) score, myeloperoxidase (MPO) activity, and colon tissue damage. In addition, EPS-1 administration significantly decreased the levels of pro-inflammatory cytokines and increased levels of anti-inflammatory cytokines. Meanwhile, EPS-1 administration significantly up-regulated the expression of tight junction proteins and mucin. Furthermore, EPS-1 administration modulated gut microbiota composition caused by DSS and increased the short-chain fatty acids (SCFAs) levels. Collectively, our study showed the alleviative effects of EPS- isolated by *L. helveticus* KLDS1.8701 on DSS-induced UC via alleviating intestinal inflammation, improving mucosal barrier function, and modulating gut microbiota composition.

## 1. Introduction

Ulcerative colitis (UC) is a non-specific chronic inflammatory disease with lesions located in the colon and rectum. The typical characteristics of UC are bloody diarrhea, abdominal pain, weight loss, and ulcer [1]. The etiological mechanism of UC is complex, and current studies suggest that its pathogenesis may be related to environmental factors, genetic susceptibility, epigenetic modifications, intestinal mucosal barrier damage, intestinal microecological disorders, and intestinal immune response abnormalities [2,3,4]. The intestinal barrier has the function of absorbing and digesting nutrients and resisting invasion of foreign harmful substances [5]. Previous studies have shown that excessive apoptosis of intestinal epithelial cells will destroy the intestinal epithelial barrier and lead to the development of ulcerative colitis [6]. Gut dysbiosis has been linked with intestinal mucosal changes and mucosal inflammation. Consequently, postulations that the gut microbiome constituents can either improve or worsen UC conditions have been made [7,8]. The immune response and inflammatory pathway of UC have shown that tissue damage is driven by dynamics and complexes of cells and cytokines [9]. Traditional drugs for the treatment of UC, such as 5-aminosalycilates, glucocorticoids, immunosuppressants, and biological preparations can induce mucosal healing and alleviate the clinical symptoms of patients to a certain extent while they are prone to relapse and have certain side effects. Therefore, it is necessary for us to seek some new, low-toxic, safer, and more efficient agents.

Currently, there has been an increasing interest in exploiting the health-promoting properties of the lactic acid bacteria (LAB)-produced exopolysaccharide (EPS) in aspects such as immune-stimulatory, cholesterol-lowering, anti-diabetic, and antimicrobial activities [10,11,12]. Likewise, the anti-inflammatory potential of EPS has been widely studied in recent years [13]. EPS produced by *Streptococcus thermophilus* could alleviate the intestinal inflammation and improve mucosal barrier in the Caco-2 monolayer and the dextran sulphate sodium (DSS)-induced UC mouse model [14]. Yan et al. have illustrated that the EPS produced by *Bifidobacterium longum* subsp. *longum* YS108R could protect against the development of UC by maintenance of the mucosal barrier and gut microbiota modulation [15]. The EPS produced by *L. plantarum* NCU116 could regulate the barrier function, modulate the gut microbiota structure, and elevate c-Jun/Muc2 signaling pathway in an UC mouse model induced by DSS [16,17]. Compared to the traditional drugs, LAB-produced EPS would likely possess beneficial effects and further avoid the severe side effects. Therefore, EPS of LAB have been developed as food additives or functional food ingredients for their commercial exploitation, especially with health benefit purposes [18].

*Lactobacillus helveticus* KLDS1.8701 was isolated from traditional fermented dairy product in Sinkiang Province, China, and has recently been shown to have the capacity to effectively alleviate diarrhea in mice via the modulation of gut microbiota and the function of improving immune system. Furthermore, EPS-1 produced by *L. helveticus* KLDS1.8701 could mitigate oxidative stress via manipulating the gut microbiota composition [19]. The aim of this study was to evaluate the anti-inflammatory effects of EPS isolated by *L. helveticus* KLDS1.8701 on the DSS-induced UC model.

## 2. Materials and Methods

### 2.1. Bacteria Growth Condition, Isolation and Purification of EPS

*L. helveticus* KLDS1.8701 was isolated from traditional fermented dairy products in Sinkiang, China, and was identified by 16S rDNA similarity analysis. The strain was deposited in the Key Laboratory of Dairy Science (KLDS) of the Northeast Agricultural University (NEAU), Ministry of Education in China. The bacterial strain was cultured in the de Man, Rogosa and Sharpe (MRS) medium at 37 °C for 24 h and was sub-cultured twice prior to the experiment. EPS was extracted as previously described [19].

### 2.2. Animals and Experimental Design

A total of 36 male C57BL/6 mice (7 weeks old) were supplied by Beijing Vital River Laboratory Animal Technology Co., Ltd. (Beijing, China) and maintained under standard laboratory conditions: temperature (22 ± 2 °C), humidity (50 ± 10%), and light (12 h light/dark cycle). All animal experiments were approved by the Northeast Agricultural University animal care and welfare committee (ethic approval code: NEAUEC2001121). After 7 days of acclimatization, all mice were randomly and equally divided into three groups (*n* = 12). From days 1 to 21, the control group (NC) and the DSS intervention group (DSS) were orally gavaged with sterile PBS (1 mL/100 g body weight/day). The EPS-1 group was given EPS-1 produced by *L. helveticus* KLDS1.8701 (200 mg per kg body weight). Except for the NC group, the other two groups were induced from days 15 to 21 with 3.0% (*w/v*) DSS.

At the end of the experiment, the mice were anesthetized and operated with a cardiac puncture to collect blood. The supernatant serum was obtained by centrifuging (3000 rpm, 20 min, 4 °C), followed by storage at −80 °C. The colon part from the caecum to the anus was removed to measure its length. Then, the colon was washed with ice-cold saline and cut into two parts: one half was stored at −80 °C for biochemical assays, and the other was fixed in 4% paraformaldehyde for histological examination.

### 2.3. Assessment of Colitis

During the experiments, the body weight, stool characteristics, and bloody feces were measured daily. The disease activity index (DAI) was measured by combining (i) body weight loss (0: no loss, 1: 0–10%, 2: 10–15%, 3: 15–20%, 4: >20%), fecal status (0, normal stools; 1: loose stools (not attached to the anus); 2: loose stools (attached to the anus); 3: diarrhea (liquid) and 4: severe diarrhea), and bloody stools (0: negative fecal occult blood; 2: positive fecal occult blood; and 4: visible rectal bleeding) [20]. The myeloperoxidase (MPO) levels of supernatant were measured using the Myeloperoxidase Test Kit (Nanjing Jiancheng Co., Ltd., Nanjing, China). The length of the colon was measured by a ruler.

The distal colon samples were fixed in 4% paraformaldehyde for 24 h, and the colon tissues were embedded in paraffin and stained with hematoxylin and eosin (HE) as previously described [21]. The sections were scanned by a digital scanning system and electronic sections were stored and analyzed on the computer.

### 2.4. Measurement of Cytokines in Colonic Tissues

The colon tissues were homogenized and centrifuged at 4 °C for 10 min at 13,000 rpm, and the supernatant was collected. The levels of TNF-α, IL-1β, IL-6, and IL-10 in the supernatant were measured using ELISA kits (Nanjing Jiancheng Bioengineering Institute, Nanjing, China) following the manufacturer’s protocol.

### 2.5. Quantitative Real-Time Polymerase Chain Reaction

The total RNA of colon tissue was extracted using the TRIzol reagent (Life Technologies, Carlsbad, CA, USA) and total RNA was used to synthesize cDNA using the reverse transcribed kit (TransGene Biotech, Beijing, China). Then, a quantitative real-time polymerase chain reaction (qRT-PCR) was performed by using the SYBR Green Kit (Tiangen Biotech Co., Ltd., Beijing, China). The primers of zona occludens 1 (ZO-1), Occludin, Claudin1, Mucin 2 (MUC2) and β-actin (Sangon Biotech Co., Ltd., Shanghai, China) used in this study are shown in Table S1. The housekeeping gene β-actin was used for normalization and was calculated using the 2^−ΔΔCT^ method.

### 2.6. Gut Microbiota Analysis

The colon microbiota genomic DNA in each group was extracted using the E.Z.N.A.^®^ Stool DNA Kit (Omega Bio-Tek, Norcross, GA, USA) according to the manufacturer’s recommendations. The V3-V4 region of the bacterial 16S rDNA was amplified by PCR using the 338F and 806R primers. The raw date was merged with Flash (V1.2.11) software and filtered by QIIME (V1.9.1) to collect the high-quality clean tags [22,23]. The effective tags were clustered by UCLUST (version 1.2.22) into OTUs of ≥ 97% similarity [24]. OTUs were analyzed based on the Greengenes database by PyNAST software (Version 1.2) and were annotated with taxonomic information at the phylum and genus levels [25].

### 2.7. Short-Chain Fatty Acids (SCFAs) Analysis

Cecal contents (80 mg) were treated with the HALO-F100 fecal treatment instrument and 500 μL were taken out, which were mixed with a crotonate monophosphate solution. The supernatant was filtered through a 0.22 μm filter membrane, was transferred to a meteorological bottle and was detected by gas chromatography (GC) with an Agilent DB-FFAP capillary column (30 m × 0.25 mm ID × 0.25 μm) and an FID detector at 250 °C. The parameters were set as follows: injection volume 1 μL, split ratio 5:1, N_2_ as the carrier gas, flow rate 2.5 mL/min.

### 2.8. Statistical Analysis

Statistical analysis was performed by using one-way analysis of variance (ANOVA) with the GraphPad Prism 8.0.2 statistical software. All data were presented as means ± standard deviation (SD). For all analyses, *p* < 0.05 were considered statistically significant.

## 3. Results

### 3.1. Effect of EPS-1 Administration on the DSS-Induced Colitis in Mice

**Figure 1:**
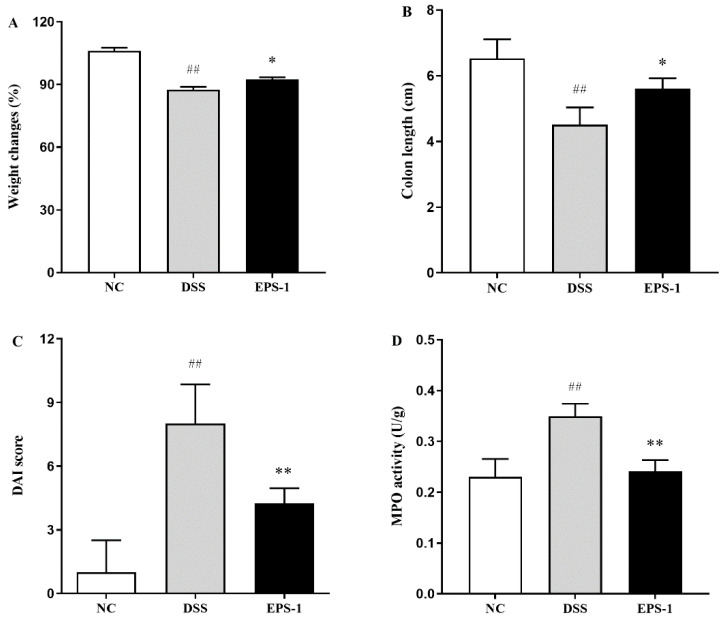
Effect of EPS-1 administration on DSS-induced colitis symptoms. (**A**) Changes in body weight (%), (**B**) Colon length (cm), (**C**) DAI score, and (**D**) MPO activity (U/g). Data are presented as mean ± SD. ^##^ *p* < 0.01 vs. the NC group, * *p* < 0.05 and ** *p* < 0.01 vs. the DSS group by using one-way analysis of variance, followed by the Duncan’s test.

As shown in Figure 1, the weight had changed, and the colon length of the mice was significantly decreased (*p* < 0.01). The DAI and MPO were significantly increased (*p* < 0.01), stimulated by DSS. Interestingly, these indexes were significantly reversed (*p* < 0.05) after EPS-1 administration, indicating that EPS-1 administration could effectively alleviate UC induced by the DSS.

### 3.2. Effect of EPS-1 on Colon Histopathological Alterations

**Figure 2:**
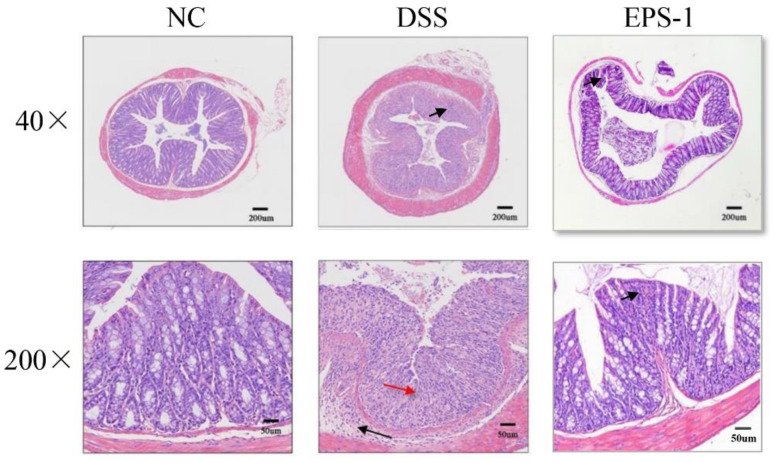
Effect of EPS-1 administration on the mouse colon histological changes (Representative hematoxylin and eosin staining).

The histological changes in each treatment group were shown in Figure 2. The colon tissue of the NC group showed complete goblet cells and intact epithelial tissue, but the crypt structure and goblet cells of the colon tissue of the mice in the DSS group disappeared, and the inflammatory cells infiltrated. After being treated with EPS-1, the colonic tissues showed improved structural damage and reduced inflammatory cell infiltration.

### 3.3. Effect of EPS-1 Administration on Inflammatory Cytokines

**Figure 3:**
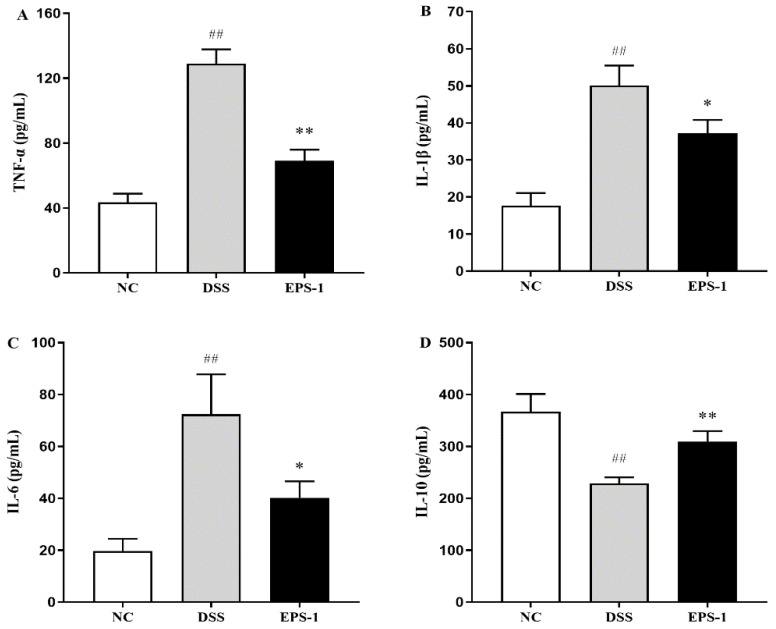
Effects of EPS-1 administration on inflammatory cytokines concentration. (**A**) TNF-α, (**B**) IL-1β, (**C**) IL-6, and (**D**) IL-10. Data are presented as mean ± SD. ^##^ *p* < 0.01 vs. the NC group, * *p* < 0.05 and ** *p* < 0.01 vs. the DSS model group by using one-way analysis of variance, followed by Duncan’s test.

The levels of inflammatory cytokines in the colonic tissue are shown in Figure 3. The levels of pro-inflammatory cytokines (TNF-α, IL-1β and IL-6) significantly (*p* < 0.01) increased in the MC group than that in the NC group, while the levels of TNF-α, IL-1β, and IL-6 of the EPS-1 group were lower than that of the MC group (*p* < 0.05). The IL-10 level in the MC group was significantly lower than that of the NC group (*p* < 0.01). Conversely, the levels of IL-10 were significantly (*p* < 0.01) reversed by EPS-1 administration. These results suggested that EPS-1 could regulate the levels of cytokines in colonic tissues of DSS-induced UC mice.

### 3.4. Effect of EPS-1 Administration on the Composition of Intestinal Barrier

**Figure 4:**
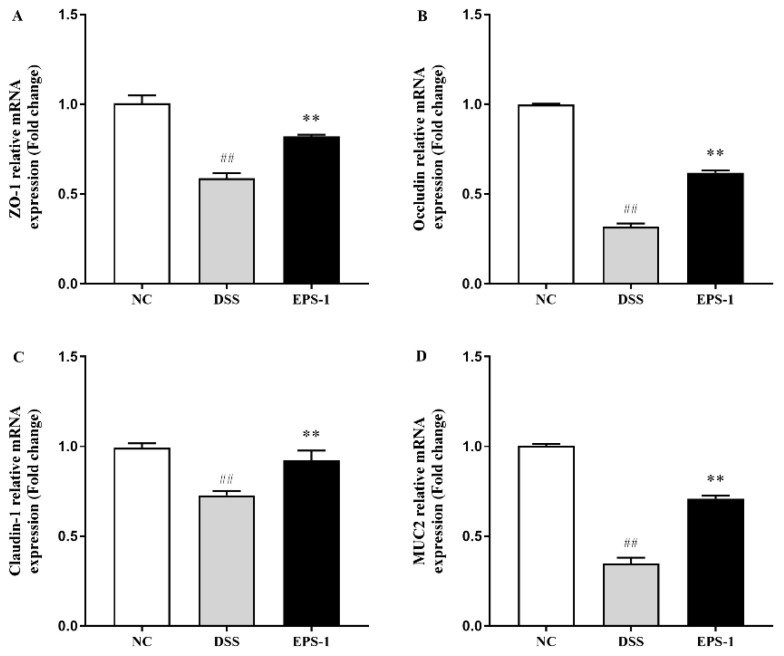
Effect of EPS-1 administration on intestinal barrier-related gene expression of colonic tissues. (**A**) ZO-1, (**B**) Occludin, (**C**) Claudin-1, and (**D**) MUC2. Data are presented as mean ± SD. ^##^ *p* < 0.01 vs. the NC group, ** *p* < 0.01 vs. the DSS group by using one-way analysis of variance, followed by Duncan’s test.

The tight junction proteins (Claudin-1, Occludin, and ZO-1) in the colon are shown in Figure 4. Compared with the NC group, the expression levels of ZO-1, Occludin, Claudin-1 and MUC2 in the MC group were significantly decreased (*p* < 0.01), implying that the epithelial integrity has been impaired. Compared with the MC group, the expression levels of ZO-1, Occludin, Claudin-1, and MUC2 were significantly reversed by the EPS-1 administration (*p* < 0.05). The finding indicated that EPS-1 administration could protect the epithelial integrity of DSS-induced UC mice.

### 3.5. Effect of EPS-1 Administration on the Composition of Gut Microbiota

**Figure 5:**
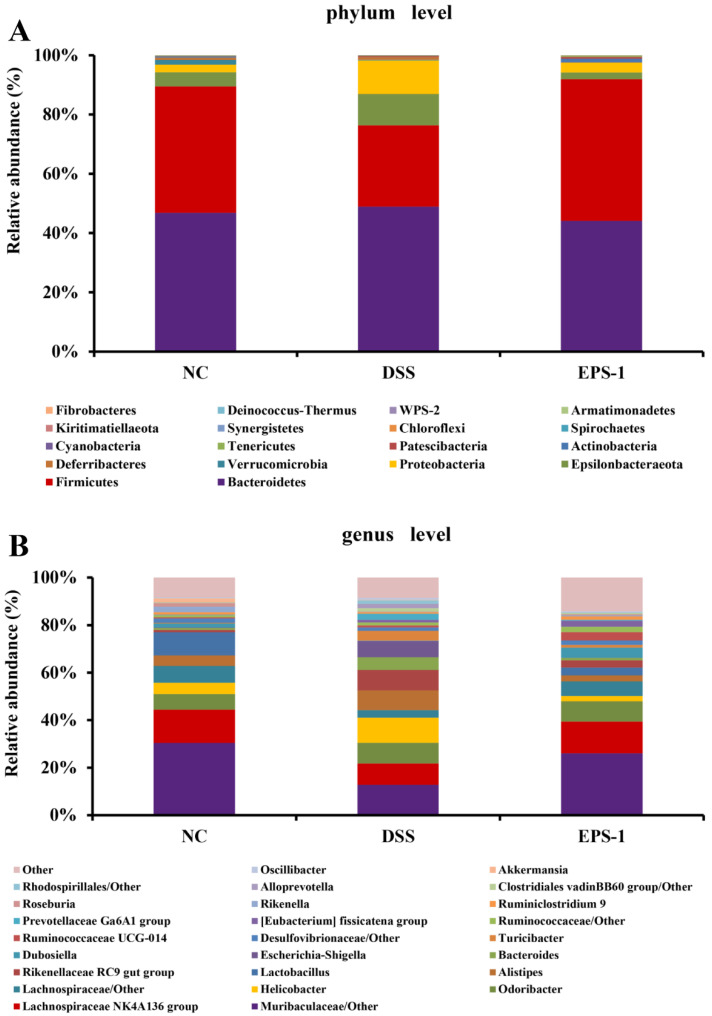
Relative abundance of microbial species at the phylum level (**A**), genus levels (**B**) in the feces of mice.

The gut microbial composition at phyla and genera levels in three groups is shown in Figure 5. At the phylum level (Figure 5A), Bacteroidetes and Firmicutes were the most abundant phyla. The relative abundances of Bacteroidetes, Epsilonbacteraeota, and Proteobacteria were higher in the DSS group than those in the NC group. Decreases in the relative abundance of Firmicutes and Verrucomicrobia were found in the DSS group when compared to the NC group. However, the EPS-1 administration partly reversed these trends. At the genus level (Figure 5B), the abundances of *Lactobacillus, Parvibacter, Enterorhabdus,* and *Alloprevotella* were decreased in the DSS group compared to that of the NC group. In addition, the relative abundances of *Helicobacter*, *Alistipes*, *Bacteroides,* and *Escherichia-Shigella* were significantly increased in the DSS group compared to that of the NC group. Interestingly, EPS-1 administration restored these changes, indicating that EPS-1 administration could modulate the gut microbiota composition of DSS-induced UC mice.

### 3.6. Effect of EPS-1 Administration on SCFAs Production

**Figure 6:**
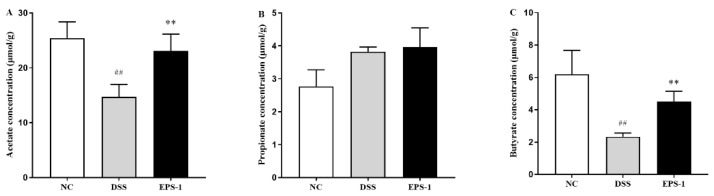
Effect of EPS-1 administration on SCFAs in contents. (**A**) acetate, (**B**) propionate, and (**C**) butyrate. Data are presented as mean ± SD. ^##^ *p* < 0.01 vs. the NC group, ** *p* < 0.01 vs. the DSS group by using one-way analysis of variance, followed by Duncan’s test.

As shown in Figure 6, the levels of acetate and butyrate were significantly decreased (*p* < 0.01) in the colon content of DSS-stimulated mice, compared with the NC group. EPS-1 administration significantly increased (*p* < 0.01) the level of acetate and butyrate compared with the DSS group. However, there were no differences of propionate among all groups. The finding indicated that EPS-1 administration could increase the level of SCFAs in colon content of DSS-induced UC mice.

## 4. Discussion

Ulcerative colitis is one of the chronic inflammatory bowel diseases caused by multiple factors with unknown causes. In recent years, incidences of ulcerative colitis have increased significantly, and the disease population tends to be younger. The World Health Organization recognizes it as one of the refractory diseases and prone to recurrent attacks [26,27,28]. At present, there is no effective and suitable drug for the treatment of UC. However, LAB-produced EPS in the treatment of UC has attracted more and more attention. Therefore, we studied the protective effects and the potential mechanisms of EPS-1 isolated by *L. helveticus* KLDS1.8701 on DSS-induced UC mice.

The DSS-induced UC mouse model has been widely used in the study of the pathophysiological mechanism of UC and the screening of potential natural active substances for treatment strategies [29]. According to reports, DSS-treated mice exhibited typical characteristics, such as weight loss and shortened colon. DAI and MPO are widely used to evaluate UC conditions [30,31,32]. DAI is composed of body weight change, diarrhea, and hematochezia and MPO is closely associated with inflammatory response and tissue damage in acute or chronic intestinal inflammation [33]. The present study found that DSS exposure for seven days could significantly decrease the weight changes and colon length and increase the DAI and MPO. However, we found that EPS-1 administration can significantly reverse these indicators. In addition, DSS-induced pathological damage in the colon was also improved after EPS-1 administration. Similar effects were also found in the previous results [14,17]. Therefore, EPS-1 administration showed a good preventive effect on the DSS-induced UC.

Numerous studies have shown that abnormal activation of intestinal immune cells is accompanied by excessive secretion of pro-inflammatory cytokines, leading to inflammation of the colon [34]. Therefore, inhibiting the secretion of pro-inflammatory factors is essential for the treatment of UC. The present study found that DSS exposure for seven days could significantly increase pro-inflammatory cytokines’ levels (TNF-α, IL-1β, and IL-6) and decrease the level of anti-inflammatory cytokine (IL-10). TNF-α is a key pro-inflammatory factor that can have a synergistic effect with a variety of cytokines to further induce the release of inflammatory mediators in the body. In UC patients, TNF-α can also destroy intestinal epithelial cells, induce colonic epithelial cell apoptosis, and cause intestinal mucosal damage. Excess IL-1β promoted the expression of other inflammatory factors, enhanced the permeability of endothelial cells and epithelial cells, and aggravated the inflammation of intestinal mucosa. IL-6 is a pro-inflammatory factor, which plays an important role in the process of inflammatory response and immune regulation. IL-10 is an anti-inflammatory factor that inhibits the release of pro-inflammatory factors and reduces the inflammatory response [35]. These inflammatory cytokine levels were significantly regulated by EPS-1 administration; a similar result was found in the study of Zhang et al. [36]. These findings suggested that EPS-1 had good anti-colitic effects by adjusting the levels of cytokines in colonic tissue.

During the occurrence and development of colitis, the reduction of tight junction proteins often leads to an increase in intestinal permeability and damage to the intestinal barrier, which will expose the immune cells of intestinal antigens to the mucosa and submucosa, and ultimately lead to inflammation reaction [37]. Therefore, maintaining the integrity of intestinal barrier function and structure is essential to preventing the occurrence and development of intestinal inflammation. DSS-induced colitis disrupts the integrity of the intestinal barrier in mice, whose epithelial barrier structure is typically disrupted by tight junction proteins and altered intestinal permeability. The mRNA expression levels of Claudins, Occludin, ZO-1, and MUC2 proteins in the DSS group were significantly decreased compared with those in the NC group. ZO-1 plays an important role in coordinating cell bypass barrier function and balancing intestinal epithelial cell permeability [38]. Occludin is a kind of overall transmembrane protein, which is one of the most important protein molecules in tight junctions [39]. Claudins play a key role in intestinal epithelial homeostasis and the expression and regulation of inflammation [40]. Mucins mainly include MUC1-MUC6, among which the mucus layer in the colon is the main MUC2 [41]. The lack of tight junctions will increase the permeability of the intestinal barrier, resulting in the invasion of bacteria and potentially harmful antigens, and then triggering and promoting the occurrence of intestinal inflammation [42]. The mRNA expression levels of these genes related to intestinal barrier function were significantly elevated by EPS-1 administration, which were in accordance with the previous study [16]. These findings indicated that EPS-1 administration could improve the intestinal barrier function disrupted by DSS.

Accumulating studies reported that dysbiosis of the gut microbiota was observed in IBD. Gut dysbiosis has been linked with intestinal mucosal changes and mucosal inflammation. This has led to postulations that gut microbiota composition can either ameliorate or aggravate IBD symptoms [43,44]. In this study, the relative abundances of Bacteroidetes, Epsilonbacteraeota, and Proteobacteria were higher in the DSS group than those in the NC group, which is consistent with the previous studies [21,45]. In addition, the increase in the abundance of Proteobacteria is regarded as a microbial signature of gut microbiota imbalance [46]. The abundances of *Lactobacillus, Parvibacter, Enterorhabdus,* and *Alloprevotella* were decreased in the DSS group compared to that of the NC group. In addition, the relative abundances of *Helicobacter*, *Alistipes*, *Bacteroides,* and *Escherichia-Shigella* were significantly increased in the DSS group compared to that of the NC group. *Helicobacter* and *Escherichia-Shigella* are gram-negative bacteria that can damage the immune system and aggravate intestinal infections, which is usually related to the pathogenesis of UC [47]. It has been reported that *Bacteroides* and *Alistipes* genera can secrete LPS [48]. Recent studies found that probiotics and prebiotics administration increased the levels of *Lachnospiraceae NK4A136 group* caused by DSS, which produces SCFAs and plays a critical role in relieving colitis [49]. Interestingly, EPS-1 administration restored the changes compared to what was observed in the DSS group. These results indicated that EPS-1 administration could alleviate DSS-induced colitis through restoring the gut microbiota composition.

SCFAs have been demonstrated to exert beneficial functions in several diseases, such as diabetes, IBD, and cancer 51], due to the ability to enhance intestinal barrier, reduce inflammation, and promote epithelial cell proliferation [50,51]. SCFAs possessed the positive effect on ameliorating UC. Therefore, SCFAs in colon content were measured in our study to evaluate the effect of EPS-1 administration on suppressing DSS-induced colitis. The levels of acetate and butyrate were significantly decreased in the colon content of DSS-stimulated mice, compared with the NC group. EPS-1 administration significantly increased the level of acetate and butyrate compared with the DSS group. Tayyeb et al. demonstrated that SCFAs reduced pro-inflammatory cytokines via mediating NF-κB inhibition [52]. The finding is consistent with the results that EPS-1 administration significantly reduced the pro-inflammatory cytokines. Tong et al. reported that SCFAs improved intestinal barrier function via the STAT3 signaling pathway to increase ZO-1 and Occludin expression in the DSS-induced colonic tissue [53]. The finding is in accordance with the result that EPS-1 administration significantly improved the intestinal barrier integrity mentioned above. These findings indicated that EPS-1 administration could ameliorate DSS-induced colitis by increasing the levels of SCFAs in colonic content.

In a word, this study preliminarily found that EPS-1 administration regulated gut microbiota and their metabolites to attenuate UC. However, the underlying mechanism has not been explored at the molecular level.

## 5. Conclusions

EPS-1 could alleviate DSS-induced colitis in mice by regulating inflammatory cytokines, protecting intestinal barrier function, modulating the gut microbiota structure and increasing the SCFAs level. We will focus on how the SCFAs affected the expression level and post-translation modification of the anti-inflammatory signaling pathways in a future study.

## Supplementary Materials

The following are available online at https://www.mdpi.com/article/10.3390/microorganisms9102086/s1, Table S1: Sequences of primers used for qPT-PCR.
