# Supplementary material for: Alleviative Effects of Exopolysaccharide Produced by Lactobacillus helveticus KLDS1.8701 on Dextran Sulfate Sodium-Induced Colitis in Mice
Source: Microorganisms. 2021 Oct 2;9(10):2086. doi: 10.3390/microorganisms9102086 (PMC8539248; doi:10.3390/microorganisms9102086)
Supplement: Supplementary file 1 [file microorganisms-09-02086-s001.zip › microorganisms-1376222-supplementary.pdf]

Table S1 Sequences of primers used for RT-qPCR

Gene	Forward primer sequence (5′-3′)	Reverse primer sequence (5′-3′)
ZO-1	GCGAACAGAAGGAGCGAGAAGAG	GCTTTGCGGGCTGACTGGAG
Occludin	TGGCTATGGAGGCGGCTATGG	AAGGAAGCGATGAAGCAGAAGGC
Claudin1	GCTGGGTTTCATCCTGGCTTCTC	CCTGAGCGGTCACGATGTTGTC
MUC2	TGCTGACGAGTGGTTGGTGAATG	TGATGAGGTGGCAGACAGGAGAC
β-actin	GGTTGTCTCCTGCGACTTCA	TGGTCCAGGGTTTCTTACTCC
